# Nonmedical prescription psychiatric drug use and the darknet: A cryptomarket analysis

**DOI:** 10.1016/j.drugpo.2019.01.016

**Published:** 2019-11

**Authors:** Jack Cunliffe, David Décary-Hêtu, Thomas A. Pollak

**Affiliations:** a School of Social Policy, Sociology and Social Research, University of Kent, United Kingdom; b École de Criminologie, Université de Montréal, Canada; c Department of Psychosis Studies, Institute of Psychiatry, Psychology and Neuroscience, King’s College London, United Kingdom

**Keywords:** Digital trace methodology, Cryptomarkets, Psychiatric drugs, Sedatives, Stimulants, Opioid dependency, Darknet markets, Nonmedical prescription drugs

## Abstract

**Background:**

Nonmedical prescription psychiatric drug use (NMPDU) is an increasing global health problem, with recent concern focusing on darknet cryptomarkets as sources of procurement. There is a shortage of evidence regarding comparative worldwide NMPDU trends, due in part to data collection difficulties. This problem is particularly marked for non-opioid drugs, particularly those psychiatric drugs which act on the central nervous system (CNS) and have high misuse potential and are associated with high levels of dependency and fatal overdose. This paper therefore has two goals: 1) to report on the kinds of psychiatric prescription drugs available on cryptomarkets, and 2) to use this data to uncover temporal and geographical trends in sales of these products, potentially informing policy regarding NMPDU more generally.

**Method:**

Digital trace data collected from 31 cryptomarkets in operation between September 2013 and July 2016 was analysed by country of origin descriptively and for trends in the sales for 7 psychiatric drug groupings, based on their main indication or intended use in psychiatric practice.

**Results:**

Sedatives (such as diazepam and alprazolam) and CNS stimulants (mainly Adderall, modafinil and methylphenidate) had the greatest share of sales, but usage and trends varied by location. The UK has high and rising levels of sedative sales, whilst the USA has the greatest stimulant sales and increasing sedative rates. Sales of drugs used in the treatment of opioid dependency are also substantial in the USA. The picture is less clear in mainland Europe with high sales levels reported in unexpected Central and Northern European countries. There is evidence of a move towards the more potent sedative alprazolam – already implicated as a source of problematic NMPDU in the USA – in Australia and the UK. Sales of drugs such as antidepressants, antipsychotics, mood stabilisers and antidementia drugs – all drugs with limited abuse potential – were negligible, indicating minimal levels of online cryptomarket procurement for self-medicating mental health problems.

**Conclusion:**

Predominantly, psychiatric drugs with potent sedative, stimulant or euphoriant effects are sold on cryptomarkets and this varies by country. With some caveats regarding the limitations of cryptomarket digital trace data taken into account, the study of trends of these products sold online over time may offer a novel and increasingly important window onto wider drug purchasing habits.

## Background

### Nonmedical use of prescription drugs: an emerging global health and policy challenge

Nonmedical prescription drug use (NMPDU) represents one of the biggest public health challenges facing the world today. Reasons for NMPDU are highly variable and to some extent differ according to the class of drug concerned. These may include: the euphoriant, tension-reducing or relaxing effects of the drug; self-medication for a diagnosed, undiagnosed or self-diagnosed mental or physical health problem; performance enhancement (e.g. in an academic context); or enhancement of the effects of drugs of abuse taken concomitantly (Mccabe, Boyd, & Teter, 2009). NMPDU is associated with substance abuse disorders (Boyd, West, & Mccabe, 2018; McCabe, Teter, Boyd, Wilens, & Schepis, 2018) and a range of other psychiatric outcomes including depression and suicidality (Mclarnon, Monaghan, Stewart, & Barrett, 2011; Schepis, Teter, Simoni-Wastila, & Mccabe, 2018). Rates of NMPDU are increasing throughout the world, and have been associated with increases in drug-related hospital attendances and deaths. In Europe in 2016, 21% of drug-related emergency hospital presentations were associated with the misuse of prescription or over-the-counter drugs (mainly benzodiazepines and opioids) (European monitoring centre for drugs & drug addiction, 2018).

While attention has focused on the nonmedical use of opioids (Haffajee & Frank, 2018; Martin, Cunliffe, Decary-Hetu, & Aldridge, 2018), there is an emerging recognition that use of other medication classes represents a major component of NMPDU. In particular, nonmedical use of sedative medication, mainly of the benzodiazepine class, has received increasing recent media coverage, with alprazolam (sold under the trade name Xanax) singled out due to its alleged recent glamorisation in Hip Hop culture in the USA and its links to the death of a number of well-known artists (Beaumont-Thomas, 2017). The benzodiazepines alprazolam and diazepam are among the ten drugs (prescription and non-prescription/illicit) most frequently implicated in drug overdose deaths in the USA, with alprazolam consistently outranking even methamphetamine (Warner, Trinidad, Bastian, Minino, & Hedegaard, 2016). In the UK, clinical services have warned of a rise in young people seeking help for problems with NMPDU, with the Central and North-West London NHS Foundation Trust opening a specific Addiction to Online Medicine (AtOM) treatment clinic in 2018, with a specific concern for benzodiazepine and, particularly, alprazolam abuse (Marsh, 2018). In January 2018, the UK government launched a review of evidence on the scale and nature of nonmedical prescription medicine use, due to publish in early 2019 (Public Health England, 2018) - a move that followed similar consultation by the Advisory Council on the Misuse of Drugs recommendation (Iversen, 2016b; Iversen, 2016a) that gabapentoids –which are prescribed for the treatment of neuropathic pain, anxiety or seizures – be scheduled under the 2001 Misuse of Drugs Regulations due to increasing nonmedical usage (a move that was subsequently appended as a recommendation in October 2018). The European Drug Report 2018 identified the increasing use and availability of sedative drugs amongst young people as an area of particular concern, requiring further investigation, policy consideration and prevention efforts. Since 2014, 14 new benzodiazepines have been reported to the EU Early Warning System, indicating substantial efforts towards illicit drug development within this drug class (European monitoring centre for drugs & drug addiction, 2018). In the USA, benzodiazepine prescription rates continue to increase (Bachhuber, Hennessy, Cunningham, & Starrels, 2016; Lembke, Papac, & Humphreys, 2018). While rates appear to have fallen in the UK, a close inspection of the data suggests that this is in large part driven by falling prescription rates of temazepam (listed as a Schedule 3 controlled drug in 2015), with continuing increases in prescriptions of other benzodiazepines such as diazepam and lorazepam (Prescribing & Medicines Team Health & Social Care Information Centre, 2016).

Nonmedical use of prescription stimulant medication typically involves the use of medications prescribed for attention-deficit hyperactivity disorder (e.g. methylphenidate or Ritalin) or narcolepsy (e.g. modafinil), with consistently high rates reported among college students who report using the drugs to enhance academic performance (a 2015 meta-analysis estimated the prevalence to be 17% – Benson, Flory, Humphreys, & Lee, 2015) and recent evidence indicating use amongst school-age adolescents (Benson et al., 2015; Han, Jones, Blanco, & Compton, 2017; Striley, Kelso-Chichetto, & Cottler, 2017; Wang, Cottler, & Striley, 2015). There is a relative dearth of information about the nonmedical use of antipsychotic or antidepressant medications, classes which have little by way of immediately rewarding or stimulating properties, and for which nonmedical use is therefore likely to represent self-medication (although with some exceptions (Chiappini & Schifano, 2018)).

Direct comparison of usage rates by country is scant, despite the attention these products have received from the media internationally. Rates in the USA have received most attention and are usually drawn from the National Survey on Drug Use and Health. McCabe et al. (2018) pool data from 2009 to 2014 and report on educational differences in usage rates, finding that full-time college students and college graduates had the highest rates of prescription stimulant misuse, with around 4% having used these substances in the past year. In the EU the evidence is similarly sparse, barring some limited ECMDDA secondary data collection. Novak et al. (2016) surveyed just over 22,000 people aged between 12 and 49 in Denmark, Germany, Great Britain, Spain and Sweden with the intention of investigating NMPDU, and the characteristics of those users. They find that Spain and Sweden had the most prevalent use of sedatives, followed by Great Britain and Denmark. Germany had the lowest percentage of users in the population for both stimulants and sedatives. The work reports usage rates in EU countries slightly below those seen in the USA (20% lifetime usage vs between 7 and 13% in EU).

Studies of procurement sources of prescription drugs for nonmedical use have consistently identified procurement via a friend or family member as the most common method, followed by taking them from someone without their knowledge (Han et al., 2017) – presumably predominantly from those who had accessed them originally from a doctor. Most studies have identified procurement via the internet as being endorsed by fewer than 10% of nonmedical prescription drug users (McCabe et al., 2018; Novak et al., 2016). Online pharmacies (of either the ‘no prescription required’ or ‘free online consultation’ varieties) have been the main source of online procurement of prescription drugs since the early 2000s. Initially set up in response to spiralling medication costs in the USA, the early 2000s saw a proliferation of these vendors, both legitimate and illegitimate, often based or purporting to be based in Canada. Monitoring these ‘clear net’ vendors is challenging and legitimacy is often difficult to assess due to the short lifespan of the websites and the use of proxy servers obfuscating the true location of vendor operations (Festinger et al., 2016). Although in the USA in 2010 the internet was found to represent a negligible, and declining, source of procurement of nonmedical prescription drugs (Inciardi et al., 2010), the proliferation of clear net pharmacies has continued unabated, with the monitoring agency LegitScript estimating in 2015 (Horton, 2015) that at any one time there are 27,500 to 40,000 illegal online internet pharmacies in operation. Recent blog posts from LegitScript have focused on "How Drugs Are Sold on the Dark Web” (Khalaf, 2018) reflecting a growing concern about these avenues for acquiring prescription drugs, and possible replacement of online pharmacy sources.

### Cryptomarkets and cryptomarket research

Since the launch of Silk Road in 2011 cryptomarkets (a phrase first coined by Martin, 2014a) have grown in size and importance, with Kruithof et al. (2016) estimating them to be responsible for around $170 m per year of drugs trade, up from an estimate of $100 m in 2015 (Soska & Christin, 2015: 40), though Global Drug Survey estimates that the growth has varied by region (UNODC, 2017b). Although various law enforcement actions, closures, scams and other developments have affected the composition of the markets, with new domains appearing and disappearing relatively frequently, the basic function of these has remained broadly similar with resilient growth (Décary-Hétu & Giommoni, 2017; Van Buskirk et al., 2017). They have been described, variously, as transformative of the drug trade (Martin 2014b), a “paradigm shift in criminal innovation” (Aldridge & Décary-Hétu, 2014) or leading a gentrification of drug markets (Martin, 2018).

Following the initial June 2011 Gawker article (Chen, 2011) that brought Silk Road to the attention of the world, academic interest was quick to react. Barratt’s (2012) missive that “we should definitely watch this space” (Barratt, 2012: 107) was quickly followed by Christin’s (2013) computer science-oriented paper that first utilised what Décary-Hétu and Aldridge (2015) call the ‘digital trace’ methodology (essentially downloading and organising the content of the live online markets on a regular basis, and using this as the basis for analysis) and case studies of user experience (Van Hout and Bingham, 2013a, Van Hout and Bingham, 2013b). The field has since proliferated using an ever-diversifying array of research methodologies. These methods broadly split into 3 domains: qualitative, survey and the aforementioned digital trace methods, with a small number of researchers analysing test buys (Rhumorbarbe, Staehli, Broséus, Rossy, & Esseiva, 2016; Caudevilla et al., 2016; Van der Gouwe, Brunt, van Laar, & van der Pol, 2017; Quintana et al., 2017). Together, these works have uncovered a great deal of important data about the characteristics, day-to-day functioning and impacts of cryptomarkets, often with a focus on their transformative potential, harm reduction mechanisms, or both.

Qualitative and survey-based research has provided crucial insights into the user experience, including the factors influencing judgement of product quality (Bancroft & Reid, 2016) and the complex relationship between cryptomarket use, harm avoidance and exposure to violence (Barratt, Ferris, & Winstock, 2016, 2016). More frequently used than these methodologies, however, are the so-called ‘digital trace’ approaches. Although relatively difficult to quantify as the phrase ‘digital trace’ in not in universal usage, of the 62 studies returned from the search term “cryptomarket” on Web of Science in August 2018, 29 use some form of digital trace method – though methodologies have developed, been refined and in some cases results questioned (see Munksgaard, Demant, & Branwen, 2016). Of the remaining 33 studies, 9 provide commentaries based predominantly on digital trace work. These works have uncovered a number of insights about how cryptomarkets work, including: how competitive it is to be a successful vendor (Paquet-Clouston, Décary-Hétu, & Morselli, 2018); the usage of free samples with purchases to gain customer loyalty (Ladegaard, 2018); the growth in trade of new psychoactive substances (Wadsworth, Drummond, & Deluca, 2018); consumer loyalty to specific vendors (Décary-Hétu & Quessy-Doré, 2017); the usage of the markets to facilitate business-to-business transactions (Aldridge & Décary-Hétu, 2016; Demant, Munksgaard, & Houborg, 2018); the constrained geographical spread of the markets and within-region distribution of that trade (Broséus, Rhumorbarbe, Morelato, Staehli, & Rossy, 2017; Martin, Cunliffe, Décary-Hétu, & Aldridge, 2018; Norbutas, 2018; Tzanetakis, 2018); and the approximate value and product composition of the markets (Christin, 2013; Soska & Christin, 2015; Kruithof et al., 2016; Tzanetakis, 2018).

What is, for the main, missing from the analysis of cryptomarket data are the implications for knowledge about the wider drug trade. On the one hand this is to be expected: as a relatively new phenomenon researchers have been busy getting to grips with the methods of investigation and understanding the structures these new forms of drug distribution bring, along with the impact they have directly on users. On the other hand these markets may represent an as-yet underutilised opportunity for informing policy more widely. For example, utilising digital trace information could help inform ways of disrupting their trade from a law enforcement perspective (Broséus et al., 2016; Mireault, Ouellette, Décary-Hétu, Crispino, & Broséus, 2016), or using the data for an analysis of the structure of drug networks more generally (Duxbury & Haynie, 2017). Whilst looking at the specifics of the Australian online drug trade, Cunliffe, Martin, Décary-Hétu, and Aldridge, (2017) compared online to offline prices and found that the large differential in the methamphetamine market is indicative of the higher levels of risk through violent exchanges present in that specific market, driven in no small part by the involvement of motorbike gangs, and posed the question as to whether this differential can be used to uncover violent drug markets prospectively. In one of the only published studies to examine the phenomenon of NMPDU on cryptomarkets, Martin, Cunliffe, Decary-Hetu et al. (2018) used a similar digital trace approach to examine the impact of the 2014 rescheduling of hydrocodone combination products in the USA. They found a rise in the sales of opioids after the law change with no differences in other comparable drug categories or jurisdictions. The authors suggested this is demonstrative of the inadequacy of supply side interventions in drug markets, without the demand side also being addressed, and posit that the trend is likely indicative of a wider movement toward illegal buying of these products both on and offline.

## Research aims

This work therefore has two interwoven aims. The first is to report in detail on the nature of the non-opioid prescription medications on online cryptomarkets: how prevalent are these sales, what is their geographical distribution and how have they changed over time? This is important information and something that has not been fully focused on before. We focus on non-opioid drugs that act on the central nervous system and are commonly used in psychiatric practice, for reasons of focus, interest and that opioid rates have been reported in detail in other works (Martin, Cunliffe, Decary-Hetu et al., 2018) – although drugs used for the treatment of opioid dependency will be still be included. The majority of the work will focus on those countries that show the largest number of cryptomarket drug sales (as reported in numerous previous pieces of work) but will also focus on India and China, due to their emerging reputation as source countries for either generic (non-branded) or clandestine production of prescription drugs (Kmietowicz, 2015; UNODC, 2017a).

By situating this analysis within the wider cryptomarket literature, and with an awareness of the broader cryptomarket trading characteristics, the secondary aim of the investigation is to establish to what extent the cryptomarket data can be informative regarding the nature of the illicit market for psychiatric drugs more generally. As discussed, there is lack of information about trends in these types of drug market, and despite the increasingly panicked tone of some recent media coverage a major question is whether cryptomarket research can shine a light on the reality of usage of these products. At the most general level, it is hoped that this work can further demonstrate that, with the appropriate limitations and nuances being included, cryptomarket digital trace analysis can be used as resource to understand wider trends in drug usage rates.

## Method

The data used in this paper were obtained from the DATACRYPTO software (Décary-Hétu and Aldridge, 2013) that crawls, downloads and processes the cryptomarket HTML pages into an analysable format. This data source has been verified by an independent panel as accurate and sufficiently reliable for use in research such as the present study and has been used in numerous previous peer reviewed publications (for example: Kruithof et al., 2016; Décary-Hétu & Giommoni, 2017; Cunliffe et al., 2017; Aldridge & Décary-Hétu, 2016). The specific version of the dataset used in this paper holds information from 12^th^ September 2013 to 18^th^ July 2016, covering 31 of the largest English language markets that were in operation over that time period. Information was typically collected ever 2 weeks, with some gaps including a 45 day gap between the 30th September and the 14th November 2014, roughly coinciding but not related to Operation Ononymous (Décary-Hétu & Giommoni, 2017), and a 105 day gap between 20th March 2015 and 3rd July 2015 due to technical refinements to the software. This is the same dataset used by Martin, Cunliffe, Decary-Hetu et al. (2018) to analyse the impact of hydrocodone combination product legislative changes in the USA. At the time of drafting only one of these markets (Dream Market) was still operational, although in keeping with the findings from Décary-Hétu and Giommoni (2017) it is expected that the previously noted resilient growth has continued.

Three key pieces of information from the collected data are used to inform the analysis: the specific drug type listed, the country from which the product would be shipped from and the number of customer feedbacks that each product had received in the 30 days previous to the data collection date. This later piece of information is taken to proxy the number of sales, an established methodology (Christin, 2013; Soska & Christin, 2015; Paquet-Clouston, 2018; Aldridge & Décary-Hétu, 2014) and is expected to be a slight undercount of actual market activity (see Martin, Cunliffe, Decary-Hetu et al., 2018, for further details). Using established methods for analysis based on DATACRYPTO, the majority of the analysis presented, unless otherwise stated, was restricted to active products (those products that had received at least one feedback in the previous 30 days) to account for dormant, fake or otherwise irrelevant listings. Prices were listed in bitcoin and converted to US dollars at the time of data collection. Again in keeping with previous work, to account for occasions where vendors artificially list their products at inflated prices when out of stock, the median price across every historical listing of a specific product was used to account for this variability.

The specific drug products present in the listing were classified into 7 groupings based on the British National Formulary (BNF) legacy classification system, which classifies prescription medications according to their main indication or intended use. This allowed for classification of most medications, although in some instances where a specific medication was not included in the BNF due to it not being licensed in the UK, it was placed in the most appropriate category based on its main indication and its pharmacological similarity to other drugs within that category. The categories used for classification were: Hypnotics and anxiolytics (or “sedatives”); Central Nervous System stimulants and drugs used for attention deficit hyperactivity disorder (“CNS stimulants”); Drugs used in the treatment of opioid dependence; Antidepressant drugs; Antipsychotic drugs; Antiepileptic drugs (used in psychiatric practice as mood stabilisers); Drugs for dementia. Details of the individual drugs in each of these categories can be found in appendix Table 1.

**Table 1:** Total number of listings, active listings and sales recorded in September 2013 to July 2016 dataset by psychiatric drug categorisation (rounded to nearest 100).

	Total distinct listings		Distinct Active listing		Total Sales	
	n	% of total	n	% of total	n	% of total
Nonpsychiatric drugs	438,800	85.8%	153,300	87.4%	1,392,100	89.5%
Hypnotics and anxiolytics	50,800	9.9%	15,300	8.7%	123,400	7.9%
CNS stimulant and ADHD	15,500	3.0%	4,900	2.8%	29,000	1.9%
Opioid dependence	4,900	1.0%	1,500	0.9%	10,400	0.7%
Antidepressant	1,100	0.2%	200	0.1%	700	0.0%
Antipsychotic	300	0.1%	<100	0.0%	200	0.0%
Antiepileptic	<100	0.0%	<100	0.0%	<100	0.0%
Dementia	<100	0.0%	<100	0.0%	<100	0.0%

The dataset is used alternatively as a longitudinal dataset to uncover trends in the data and as a whole to represent total market activity. In this latter case, feedbacks that are present in the data that occurred before the previous data collection of any one specific listing are excluded from the total feedback so that these are not double counted and artificially inflate the sales of any product. When the dataset is used longitudinally, to account for the increase in the size of the markets over the time frame studied and improvements to the coverage of the DATACRYPTO, sales are presented as a percentage of the sales within any one country, following a similar methodology to Martin, Cunliffe, Decary-Hetu et al. (2018). When used in a regression context to analyse trends in sales rates over time, a smoothing function averaging the number of sales for any one product grouping over the 15 days previous to or after the data collection date is applied to control for any issues with the completeness of any one specific data collection instance. The linear regression results therefore represent the estimated percentage of sales within any one country attributable to any one drug classification (the constant) followed by an estimation of the yearly percentage point change over the study period. All regressions were run with robust standard errors using the Huber-White sandwich method to account for a lack of normality in the dependents, heteroscedasticity, and data points with high leverage owing to the nature of the data collection process. Data manipulation was conducted in MySQL with analysis in Stata13.

## Results

### Market activity

Table 1 presents descriptive analysis of the total number of listings, the number of active listings and the total sales (as proxied by feedbacks) recording the dataset overall. It is immediately clear that there is an extremely low number of listings for the antidepressant, antipsychotic, antiepileptic and dementia products, and with so few sales of each of these products these were excluded from further analysis.

With analysis left to focus on the hypnotics & anxiolytics, CNS stimulants and opioid dependency products, Table 2 presents the global market share and within country market share trends for each of these product categories for the 9 countries with the highest overall market share, plus Netherlands and China due to their theoretical importance. All other countries not listed had less than a 0.5% share of the overall market and their trade in these products can be considered negligible. The column “overall market share” for each product group shows the amount of the total sales recorded in the dataset that are attributable to sellers within each country. It is perhaps unsurprising that the USA, with its considerably larger population and more active cryptomarket trade (Martin, Cunliffe, Décary-Hétu et al., 2018) than the other countries displayed, has the largest share of each market. More surprisingly, the UK represents 31% of the hypnotics & anxiolytics market, despite having a much smaller population than the USA. This pattern is not repeated in the CNS stimulants grouping, where the UK-based sales take just 16% of the market, compared to just under 60% being from the USA. 11% of all opioid dependency products are sold from within Germany, in contrast to the pattern seen in other two groupings where German sales are relatively small components of the market. Vendors in the Netherlands, a substantial source country for cannabis, MDMA-type products, cocaines, methamphetamine and opioids, seem to shun trade across all three of these product groups.

**Table 2:** Overall market share and within country proportion and change, September 2013 to July 2016.

	Hypnotics and anxiolytics					CNS stimulant and ADHD					Opioid dependence				
	Overall market share (number of sales)	Within country share and change				Overall market share (number of sales)	Within country share and change				Overall market share (number of sales)	Within country share and change			
		Base level	p	Percentage point yearly change	p		Base level	p	Percentage point yearly change	p		Base level	p	Percentage point yearly change	p
USA	41.4% (45,600)	7.7%	<0.001	0.5%	0.015	59.2% (14,600)	2.5%	<0.001	0.5%	<0.001	64.4% (5600)	1.0%	<0.001	0.1%	<0.001
UK	31.1% (34,300)	8.5%	<0.001	0.9%	<0.001	15.8% (3900)	2.7%	<0.001	−0.7%	<0.001	10.4% (900)	0.5%	<0.001	−0.1%	0.006
Netherlands	0.7% (800)	1.1%	0.012	−0.3%	0.181	2.8% (700)	−0.6%	0.092	1.0%	<0.001	0.9% (100)	0.1%	<0.001	0.1%	<0.001
Germany	5.1% (5600)	4.4%	<0.001	−0.9%	0.001	2.8% (700)	0.3%	<0.001	0.2%	<0.001	11.4% (1000)	0.6%	<0.001	0.0%	0.825
Australia	7.3% (8000)	7.2%	<0.001	−0.3%	0.261	10.1% (2500)	2.7%	<0.001	−0.4%	<0.001	3.5% (300)	2.6%	<0.001	−0.9%	<0.001
China	0.2% (200)	−2.0%	0.009	6.4%	<0.001	0.1% (< 100)	Less than 100 sales				No sales				
Canada	5.8% (6400)	−1.0%	0.191	6.3%	<0.001	3.7% (900)	1.5%	<0.001	−0.1%	0.074	0.4% (<100)	Less than 100 sales			
India	2.1% (2300)	24.9%	<0.001	6.3%	<0.001	3.7% (900)	14.5%	<0.001	−2.1%	<0.001	0.3% (<100)	Less than 100 sales			
Sweden	2.6% (2800)	20.6%	<0.001	9.5%	<0.001	0.5% (<100)	Less than 100 sales				5.7% (500)	8.9%	<0.001	−3.9%	<0.001
Denmark	2% (2300)	56.3%	<0.001	−3.7%	0.142	0.2% (<100)	Less than 100 sales				3.1% (300)	35.4%	<0.001	−13.4%	<0.001
Austria	1.7% (1900)	34.9%	0.264	11.4%	0.386	1% (200)	−13.6%	0.056	9.5%	0.006	No sales				

The “within country share and change” columns of the table present the within country longitudinal analysis of the market activity restricted to within each country as modelled through the smoothed regression technique outlined above. The base level column gives the estimated percentage of the internal market represented by each psychiatric drug grouping at the beginning of the period, i.e. in September 2013, with the yearly change rate column representing the average percentage point change per year to the end of the data collection in July 2016. Each of these statistics is accompanied by a p-value under the null hypothesis of the level being zero in the population of all sales. In the UK, therefore, at the beginning of the study period, hypnotics & anxiolytics represented 8.5% of all UK online cryptomarket sales and this proportion increased by just under 1% per year, and this change is statistically significant at the 99.9% level. The fastest growing markets over the study period were (in reverse order) Canada, India, China and Sweden, with growth rates of 6.3, 6.3, 6.4 and 9.5 percentage points respectively. In the case of Canada and China, this demonstrates a statistically and substantively significant increase in the amount of market activity attributable to hypnotics & anxiolytics from a negligible base level, and hypnotics & anxiolytics in Sweden and India moved from representing between a fifth and a quarter of the market to being an even larger part of their domestic markets by the end of the period.

Within the USA, CNS stimulants increased in popularity from an already high base over the time period studied, whilst the UK market shrunk relative to other drugs for sale in that region. This was also the case in India, though these products remained a substantial part of the Indian market activity. In other countries, these base levels and changes over the time period were either substantively or statistically insignificant (or both), with the exception of Austria which saw a large increase in sales of these CNS stimulants. Opioid dependency products, which as demonstrated by Table 1 represent a much smaller number of overall sales than either of the other two groupings with just over 10,000 sales across the whole time period, is heavily dominated by USA-based vendors in terms of total number of sales, with German vendors representing a further 11% of the market. Within Australia, Sweden and Denmark results should be treated with caution, as although these products appear to represent relatively large proportions of those markets, subsequent decreasing trends in this small market mean that by the end of the 2 year 10 month study period the rates of sales of these products had decreased to practically zero.

### Price analysis

The data upon which this work is based does not allow for the separating out of package sizes and the amount of active ingredient in any one sale. However given the relatively low unit price for each of these drugs, analysis of the distribution of the raw listing prices within each country can give an indication of how many doses of each product are being sold with the typical product listing (Table 3). With this caveat in mind, under the assumption of a unit price of around 2 dollars per pill, it is clear that the vast majority of listings are for more than a few doses. The median price for hypnotics & anxiolytics from the USA, for instance, at $79 implies that the most typical listing is for in excess of 20 tablets. For the two countries with the highest median priced sedatives, China and Canada, these pricing findings are strongly indicative of a business to business model similar to that identified by Aldridge and Décary-Hétu (2014).

**Table 3:** Distribution of listing prices in USD, by psychiatric drug categorisation and country of origin, active products only.

	Hypnotics and anxiolytics			CNS stimulant and ADHD			Opioid dependence		
	p10	Median	P90	p10	Median	P90	p10	Median	P90
USA	6	79	403	13	84	317	16	64	255
UK	8	32	143	8	44	167	8	39	120
Netherlands	8	47	242	15	50	161	23	90	182
Germany	16	43	165	16	61	199	27	92	299
Australia	20	60	250	13	52	244	8	27	136
China	69	500	3521	Less than 100 sales			No sales		
Canada	27	274	2373	10	32	150	Less than 100 sales		
India	37	93	319	30	83	297	Less than 100 sales		
Sweden	26	75	204	34	89	237	43	82	220
Denmark	30	73	147	29	40	55	5	5	37
Austria	21	60	115	45	128	318	No sales		

### Benzodiazepines: increasing visibility/dominance of alprazolam over time

**Fig. 1:**
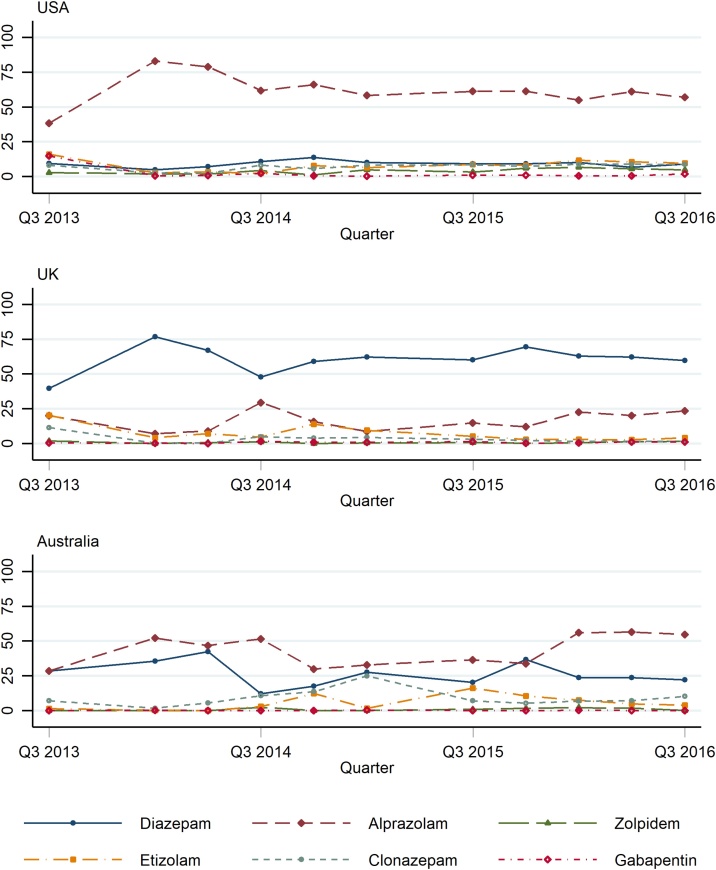
Within country percentages of hypnotic and anxiolytic sales attributable to specific drugs types, quarter 3 2013 to quarter 3 2016.

The final analytical piece is a more detailed look at the specifics within one of the drug groupings – namely the three English speaking countries with over 7% of their market represented by hypnotics & anxiolytics: the USA, the UK and Australia. Fig. 1 presents the percentage of these hypnotics & anxiolytics market sales represented by the 5 most common specific compounds within this grouping, with gabapentin included due to the ACMD (Iverson, 2016a) UK government advisory letter (pregabalin is all but completely absent from the markets with just 2 listings and zero sales). Alprazolam, more commonly known by the brand name Xanax, is by some distance the most common product in this grouping in the USA over the time frame studied, consistently representing over 50% of the market from 2014 onwards. In the UK however, where alprazolam is not approved for prescription by National Health Service (NHS) doctors, diazepam is the most common product. Towards the end of the time period there is a marked increase in the amount of the UK market represented by alprazolam. In Australia, in 2013, diazepam and alprazolam were a comparable part of the market, but by the end of the time period alprazolam had over a 50% share of this grouping’s sales.

## Discussion

One of the clearest findings from this work is one of the most simple. Although there are a range of products available on cryptomarkets across all of the drug classification groupings analysed here, it is only the products that have an abuse potential or as Novak et al. (2016) put it, that can be used to achieve a ‘euphoric state’ (we consider the term ‘pleasurable’ or ‘rewarding’ to more accurately encompass the effects of both the sedative and stimulant drug classes), that sell in appreciable quantities. This is true even if they only represent a small part of the cryptomarket trade in comparison to other products (see Soska & Christin, 2015). Products such as antidepressants, antiepileptics, antipsychotics and dementia drugs are rarely listed and even more rarely bought from these markets. This is of interest to the psychiatric profession since it suggests that many of the drug classes used in psychiatric practice are not being sourced from cryptomarkets in any meaningful amounts for the purposes of self-medication. It should however be noted that this does not imply that these products are not self-medicated or abused *at all,* merely that they are seemingly not used/abused via *purchase on cryptomarkets*. This work therefore collapses down to a study of hypnotics and anxiolytics (broadly equivalent to ‘sedatives’), stimulants and opioid dependency products. Our results are in keeping with the findings from other cryptomarket research that has taken a geographical approach (Broséus et al., 2017; Martin, Cunliffe, Decary-Hetu et al., 2018; Norbutas, 2018; Tzanetakis, 2018) showing the market is dominated by North America, Western Europe and Australia. This finding is surely demonstrative of the usage rates of cryptomarkets more generally than a reflection of the specific substances being analysed here, and once that been considered in the interpretation then the findings can indeed be used to inform policy more generally.

The USA’s dominance across all 3 drug groupings, for example, is a reflection of the usage of cryptomarkets in that country, but it is telling that whilst that country captures close to 60% of all stimulant/opioid dependency sales, when it comes to sedatives that dominancy is reduced to just over a 40% share and the UK, a much smaller country albeit one that is well represented on darknet marketplaces, accounts for close to a third of all sales – confirming the Public Health England (2018) concern that NMPDU rates are high in that country. This is seen even more clearly when considering the share of the cryptomarket trade that sedatives represented at the beginning of the study period: at 8.5% this is the highest of major cryptomarket countries (USA, UK, Netherlands, Germany and Australia – see Martin, Cunliffe, Décary-Hétu et al., 2018). Furthermore, the UK has the fastest rate of increase of those major countries – just under one percentage point per year over the study period. This should be cause for concern amongst policy makers. The analysis presented also demonstrates an increase in the USA sedative market of half a percentage point per year, indicating a general increase in that country as well, though less pronounced, again justifying wider concerns of these drugs’ usage. It is well established that Australia is a relative island when it comes to the online trade, and although hypnotics and anxiolytics represent 7.2% of all trade in that country – a cause for concern for policy makers there – there does not seem to be any change in the levels indicating the problem is likely not worsening.

When considering the sedative market in other countries, caution needs to be exercised. Norbutas (2018) found that markets are constrained by their wider geography, particularly border controls, and the findings from within the Schengen area countries are complex. Germany sedative sales are low, matching the findings of Novak et al. (2016), but claiming that this precludes there being high usage in that country would be overstating the evidence. The same can be said of the Netherlands, with its almost non-existent sedative market. Denmark and Austria both demonstrate high and consistent levels of sales for these products, and Sweden increased from a high base (over 20% of all sales) by close to 10 percentage points per year. Although Novak et al. reported high sedative usage in Sweden, for these three countries to have such exceptionally high levels is unusual and it is likely there is some cross-border/within-Schengen trade occurring.

With regard to stimulants the results clearly point to the highest usage rates being found in the USA, with other markets representing only a fraction of that share. 2.5% of the entire darknet drug market trade in that country is in these drugs, and this increased by half a percentage point per year over the 3 year study period. This is most likely a reflection of what McCabe et al. (2018) find in terms of the educational gradient for usage of these products, that those with the highest educational level have the highest usage of these products and given the widespread usage of the main components of the this category (adderall, modafinil and ritalin) as a cognitive enhancement within educational and academic settings (Benson et al., 2015; Han et al., 2017) care should be taken in educational institutions in that country to address this demand. CNS stimulants also appear to be popular in the UK and Australia, although their sales rate trends downwards in both countries, so the policy concern ought perhaps to be more muted.

Opioid dependency products are also dominated by the USA, potentially a reflection of the ongoing opioid crisis in that country coupled with their supply restrictions (Hadland & Beletsky, 2018). It should be noted that while this category does contain drugs with no inherent addictive potential (e.g. naloxone) these make up a tiny minority of all sales, which are in fact dominated by opioid drugs such as methadone and buprenorphine, drugs widely appreciated to have high diversion potential from their primary indication of managing dependence. Whilst it appears that Germany, the UK and Australia have relatively high level of these products, judged on either their market share (over 10% of overall sales for both Germany and UK) or the initial share of all online sales (2.6% in Australia at the beginning of the period, though followed by falls) these are quite low absolute numbers, with under 1000 sales in total in each country. This demonstrates a limitation of the digital trace methodology – although it appears that there is a lot of market activity in these products, with just over 10,000 sales recorded in the data, once this is split into subgroups at a country level interpretation becomes more challenging.

India, China and Canada have been carried forward in this work for theoretical reasons rather than their market size, and all three paint an interesting picture that suggests they are at least source countries, particularly of sedatives. At the beginning of the study period sedatives in India accounted for almost a quarter of all sales, and India, China and Canada all saw this part of their market grow over the time period, at just over 6% per year. This strongly suggests that there is an amount of specialisation in these products from vendors in these countries. The price analysis presented in Table 3 indicates that when these specialised vendors do sell their products, there is a tendency to sell large shipments. This is indicative of these being origin countries, with vendors able to source large numbers of these products, from either illicit production facilities (most likely in India and China) or through online pharmacies and other wholesale distribution sources (as in the case of Canada). Of course, this can only be surmised from the data; given alarming reports of contamination of illicitly manufactured benzodiazepines with other (sometimes potentially lethal) psychoactive substances, along with increasing levels of production of ‘fraudulent medicines’ (i.e. medicines that contain drugs other than labelled) (UNODC, 2017a) there is a need for research that examines the specific composition of the products bought from these countries to see what their chemical profile is and to ascertain their likely production method, potentially using the ‘test buy’ methodology alluded to in the introduction.

The final implication from this work comes from the specific analysis of the benzodiazepines comparing the UK, the USA and Australia. This paints a picture of some level of cultural transmission occurring, with an overtone of the iron law of prohibition. Alprazolam is by some way the most common sedative bought from USA vendors, whilst in the UK it shows an increasing popularity despite not being a National Institute for Health and Care Excellence (NICE) recommended drug and therefore not prescribed by NHS doctors. In Australia, too, there is an apparent movement toward this product, from a more even split between diazepam and alprazolam. There are multiple potential mechanisms driving this: there has been a sharp focus on alprazolam in US popular - particularly hip hop – culture with it being implicated in the death of Lil Peep and other musicians singing its praises (including the musician Lil Xan). On a milligram basis the potency of alprazolam is the highest of most of the commonly prescribed benzodiazepines, and this together with its short half-life is an important determinant of its abuse potential. Notably, with the exception of diazepam the other top-selling benzodiazepines – clonazepam, lorazepam and etizolam (Appendix 1) – are similarly high potency. A relationship between potency and market share (the so-called ‘iron law of prohibition’) has also been noted by Beletsky and Davis (2017) in relation to fentanyl gaining prominence in the opioid market. Of particular note in relation to sedative sales is the unexpected prominence of etizolam, a benzodiazepine derivative that is only licensed in Japan, Italy and India which has seen a steep rise in usage (and associated mortality) in the USA and Western European countries (where it largely unlicensed) in recent years (O’Connell et al., 2015; O’Connor, Torrance, & Mckeown, 2016; Iversen, 2016b), earning it the scrutiny of World Health Organisation surveillance in 2015 (WHO, 2015).

This work raises a number of policy and clinical implications: it illustrates the high level of sales in the USA for all three of the main drug categories studied here, and by implication tells a story of popular usage in that country; it shows that sedatives are an increasing issue in the UK, though CNS stimulants are less of a problem; that China, India and Canada are highlighted as problematic source countries of these products; and that there appears to be a move in the UK and Australia to more potent, less traditionally prescribed forms of sedative drug. Clinically, a clear implication of the current work is that healthcare professionals should routinely ask about the darknet as a potential source of procurement of both prescription and nonprescription drugs. Furthermore, it may be incumbent on clinicians to remain educated about the changing nature of these markets and to offer advice on the potential harms involved should darknet procurement be suspected or confirmed.

There are however many limitations - the European data is hard to untangle to a national level, with surprisingly high and low rates of sales in specific jurisdictions and the analysis of opioid products was limited by sample size despite initially appearing to represent a relatively large share of the market. Overall though, it does appear that utilising cryptomarket data such as that used here can shine a light on market activity that would otherwise be hard to pin down empirically. Cryptomarkets and cryptomarket research have come a long way in a short period time, aided by large scale analysable datasets, but these come with a risk. The UNODC (2017a) report highlights polydrug use amongst people who engage in NMPDU (often combinations of sedatives with various opioids), whilst Aldridge, Stevens, and Barratt, (2018) speculated that cryptomarkets could function as a supply gateway, and once buyers are accustomed to using these sources for purchases of whichever their primary drug of choice originally was, they may experiment with other options that are equally easily available to them online. Clearly more research is required to address the reality of this possibility, cryptomarket digital trace data will only ever be able to get so far in answering these questions and some methodological issues remain – such as the possibility that using feedback to proxy sales is an undercount of unknown magnitude (although this work follows current best practice, the utility of this approach need regular revision as the markets develop). There is very much a need for triangulation of different approaches to fully understand the function of these markets and to be able to delineate the implications that arise from the impressive data they generate.

## Conflict of interest

There are no conflicts of interest.

## CRediT authorship contribution statement

**Jack Cunliffe:** Conceptualization, Data curation, Formal analysis, Investigation, Methodology, Project administration, Resources, Software, Visualization, Writing - original draft, Writing - review & editing. **David Décary-Hêtu:** Data curation, Methodology, Writing - review & editing. **Thomas A. Pollak:** Conceptualization, Investigation, Project administration, Writing - original draft, Writing - review & editing.

## Figures and Tables

**Table:** 

Grouping	Products
Hypnotics and anxiolytics	Xanax (25%), Diazepam (21%), Alprazolam (19%), Clonazepam (6%), Valium (6%), Etizolam (5%), Ambien (3%), Zopiclone (2%), Ativan (2%), Lorazepam (1%), Lyrica (1%), Flubromazolam (1%), Nitrazepam (1%), Bromazepam (1%), Klonopin (<0.5%), Diclazepam (<0.5%), Temazepam (<0.5%), Gabapentin (<0.5%), Carisoprodol (<0.5%), Promethazine (<0.5%), other products with less than 500 total sales and representing less than 0.5% of sales
CNS stimulants and ADHD	Adderall (46%), Modafinil (28%), Ritalin (13%), Methylphenidate (6%), Dexamphetamine (4%), Dextroamphetamine (1%), Armodafinil (1%), Lisdexamfetamine (1%), Waklert (1%), other products representing less than 0.5% of sales
Opioid dependence	Suboxone (29%), Methadone (25%), Subutex (25%), Buprenorphine (22%), Naloxone (<0.5%)
Antidepressant drugs	Fluoxetine, Amitriptyline, Trazodone, Sertraline, Efexor, Bupropion, Moclobemide, Cymbalta, Zoloft, Citalopram, Celexa, Mirtazapine, other products with less than 10 sales in total.
Antipsychotic drugs	Seroquel, Zyprexa, Haloperidol
Antiepileptic drugs	Lamictal, Levetiracetam, Lamotrigine
Drugs for dementia	Namenda, Donepezil

## Appendix A Table 1: Drug categorisations

Categorisation is based on the British National Formulary legacy hierarchical categorisation system. This classifies prescription medications according to their main indication or intended use in UK medical (not specifically psychiatric) practice. In some instances where a specific medication was not included in the BNF due to it not being licensed in the UK (e.g. etizolam), it was placed in the most appropriate category (i.e. ‘hypnotics and anxiolytics’) based on its main indication and its pharmacological similarity to other drugs within that category. The gabapentoid drugs pregabalin and gabapentin were a priori moved to the ‘hypnotics and anxiolytics’ grouping from the ‘antiepileptic drugs’ grouping because in psychiatric usage (and in illicit use) they are primarily used for their anxiolytic effects, contrasting with the other drugs in the ‘antiepileptic drugs’ grouping which in psychiatric practice are used as mood stabilisers in the treatment of bipolar affective disorder and related disorders. Where trade names and generic names are given they are presented in this table as separate to reflect the nomenclature on the markets, however when analysed on a product by product basis they are combined. For example, Xanax (brand) and alprazolam (generic) are combined together in Fig. 1.

The percentages of overall sales recorded in the dataset are presented for the top three categories and for the most common product within those categories only.
